# Effectiveness of Guideline-Based Clinical Decision Support Systems: Protocol for a Systematic Review

**DOI:** 10.2196/87006

**Published:** 2026-06-11

**Authors:** Berkay Newroz Aksu, Bora Gashi, Fridtjof Schiefenhövel, Martin Boeker

**Affiliations:** 1 Chair of Medical Informatics, Institute of Artificial Intelligence and Informatics in Medicine, TUM University Hospital, School of Medicine and Health, Technical University of Munich, Ismaninger 22, Munich, 81675, Germany, 49 89 4140 4320; 2 Department of Anaesthesiology and Intensive Care (AINS), TUM University Hospital, School of Medicine and Health, Technical University of Munich, Munich, Germany

**Keywords:** systematic review, clinical guidelines, clinical decision support system, CDSS, guideline formalization, Preferred Reporting Items for Systematic reviews and Meta-Analyses, PRISMA

## Abstract

**Background:**

Clinical guidelines (CGs) standardize care through evidence-based recommendations, while clinical decision support systems (CDSS) can assist in applying these guidelines to individual patients. The scientific basis for the decisions offered by decision support systems is often not explicitly stated or not clearly specified in the literature on CDSS. Therefore, a systematic examination of the literature is needed to map the current state of CDSS, with a particular focus on the integration of CGs.

**Objective:**

This study aims to systematically collect, describe, and synthesize evidence of randomized controlled studies of interventions using CDSS with a well-defined integration of evidence-based CGs and evaluating direct medical outcomes.

**Methods:**

This systematic review adheres to the PRISMA (Preferred Reporting Items for Systematic Reviews and Meta-Analyses) and PRISMA-ScR (Preferred Reporting Items for Systematic Reviews and Meta-Analyses extension for Scoping Reviews) checklists. The eligibility criteria for this review are defined using the patient, intervention, control, outcome, and study design framework, including studies involving patients with any medical condition or disease. Study interventions need to include guideline-based CDSS, encompassing all types of interventions used for treatment. Each intervention must provide a sufficiently accessible technical description, including the types of data and algorithms used for decision-support procedures. The guidelines used within these CDSS interventions must be derived from a clearly defined, evidence-based guideline development process published by a discernible guideline-producing body. Studies must use a randomized controlled study design. Only studies evaluating the effectiveness of the interventions on direct medical outcomes are included. Web of Science, including MEDLINE, and Scopus will be searched with search expressions aligned with the eligibility criteria.

**Results:**

On August 11, 2022, the initial search was conducted on Web of Science and Scopus. From a total of 6203 records, 1347 were removed prior to screening as duplicates, 2506 records were excluded during the first screening step, and 2291 were excluded during the second step. Next, 41 papers were excluded based on full-text review, and 18 papers were finally included in the review following this initial search. This review explores whether CDSS based on CGs can improve clinical outcomes, although their effectiveness may vary depending on various factors. Potential limitations, such as high study heterogeneity, have already been identified. An update of the review has been started in April 2025.

**Conclusions:**

To our knowledge, this is the first rigorous systematic review on the effectiveness of guideline-based decision support systems in which the technical integration and algorithmic embedding of CGs have been described or can be inferred from secondary literature. With this review, we aim to address this gap by providing a detailed analysis of existing research and identifying best practices, challenges, and areas for future investigation.

## Introduction

Clinical decision-making plays a vital role in ensuring optimal patient care. Physicians constantly navigate a dense field of medical knowledge, integrating new evidence into their practice. Clinical guidelines (CGs) aim to bridge this gap by systematically synthesizing best practices and recommendations for specific conditions [1]. CGs are also crucial tools for health care providers [2], offering evidence-based recommendations for diagnosing, treating, and preventing diseases. Developed through a rigorous process involving expert committees, a thorough review of scientific evidence, and extensive peer feedback [3], these guidelines aim to streamline complex medical decisions and implement scientific evidence to improve the quality of health care decisions and outcomes for both clinicians and patients [4]. Consequently, they provide standardized approaches to diagnosis, treatment, and prevention, promoting consistency and quality of care.

In the evolving landscape of health care, optimizing clinical decision-making amid complex factors is essential for enhancing patient outcomes and incorporating evidence-based medicine, despite challenges in integrating vast amounts of clinical information and tailoring care plans to individual needs [5,6]. To optimize and support clinical decision-making processes, the health care industry has increasingly turned to clinical decision support systems (CDSS) [7]. A CDSS is a software tool designed to enhance health care delivery by providing clinicians with patient-specific assessments or recommendations based on a computerized clinical knowledge base, often integrated with electronic health records [8].

CDSS are software tools designed to assist clinicians in making patient-specific decisions by providing evidence-based recommendations, especially in situations where prescribers may lack expertise [9,10]. Despite the impressive success of model-based CDSS in specific domains [11] and the increased accessibility of AI-driven tools in health care [12], some studies have highlighted limitations in their ability to consistently improve health outcomes [13]. Therefore, knowledge-based CDSS are considered highly relevant and are widely used. These systems are more readily accepted in clinical settings because of their transparent and interpretable nature, which increases trust and improves usability among health care providers [14]. A crucial aspect of effective CDSS is the integration of evidence from CGs, which encapsulates the best available evidence and expert consensus on the management of various health conditions. By embedding these guidelines within CDSS, health care providers can ensure that their clinical decisions align with the latest standards and best practices, thereby improving patient outcomes [15]. Furthermore, such CDSS can enhance consistency in care delivery, reduce medical errors, and improve efficiency by offering ready access to relevant information [16].

However, integrating CGs into CDSS is often challenging. The scientific basis for the decisions offered by decision support systems is often not explicitly stated or not clearly specified in the literature [17,18]. A significant gap remains in the literature, as most papers do not analyze how guidelines are systematically connected and technically integrated into CDSS, particularly in conjunction with direct medical outcomes. This is the case despite numerous studies having explored the application of CDSS in various clinical domains [19,20]. Current practices rarely provide standardized methodologies that are essential for integrating guidelines into CDSS [21]. Evidence on CDSS has been systematically collected in numerous systematic reviews, assessing their impact on clinical practice [15], prescribing behavior [10,19,22,23], and health care processes [23]. Although many studies highlight potential benefits, such as improved adherence to evidence-based practices [15], readiness for CDSS uptake [24], or cost-effectiveness [25], their effects on patient outcomes remain inconclusive [16,20].

To our knowledge, there has not been a rigorous systematic review on the effectiveness of guideline-based decision support systems in which the technical integration and algorithmic embedding of CGs have been described or can be inferred from secondary literature. Existing studies tend to focus on specific aspects or applications of CDSS without providing a comprehensive analysis of how CGs are embedded within these tools [24]. Consequently, there is a significant gap in understanding the methodologies used for guideline integration, the types of guidelines most commonly used, and the impact of these integrations on clinical outcomes. Therefore, a systematic examination of the literature is needed to map the current state of CDSS, with a particular focus on the integration of CGs. This review aims to fill this gap by providing a detailed analysis of existing research and by identifying best practices, challenges, and areas for future investigation.

The objective of this systematic review is to systematically collect, describe, and synthesize evidence from randomized controlled studies of interventions using CDSS with a well-defined integration of evidence-based CGs and assessing direct medical outcomes. The scope of underlying medical conditions has not been limited in this systematic review.

## Methods

### Overview

Our methodological approach adheres to the PRISMA (Preferred Reporting Items for Systematic Reviews and Meta-Analyses) [26] and PRISMA-ScR (Preferred Reporting Items for Systematic Reviews and Meta-Analysis extension for Scoping Reviews) [27] checklists, as well as the recommendations of the Joanna Briggs Institute methodology for scoping reviews. This systematic review has been registered at PROSPERO (CRD42024605679).

The authors are aware of automated tools that can be used for various tasks in the preparation of systematic reviews, especially systematic searches, literature management, data extraction, and analysis or synthesis. For the transparency of the methods and interpretability of the results of this review, we deliberately refrain from using any tools other than those mentioned in this protocol.

### Eligibility Criteria

The eligibility criteria for this review are defined using the patient, intervention, comparison, outcome, and study design (PICOS) framework:

- Patient—studies involve patients with any medical condition or disease. There are no primary criteria for patient selection based on age, gender, or specific health conditions, ensuring broad inclusion of diverse patient populations.
- Intervention—the focus is on guideline-based CDSS interventions, encompassing all types of interventions used for treatment. Each intervention must provide a sufficiently accessible technical description detailing the types of data and algorithms used for decision support procedures, allowing for critical assessment of implementation. Additionally, the guidelines used within these CDSS interventions must be derived from a clearly defined, evidence-based guideline development process published by a discernible guideline-producing body. The CGs should be accessible in English or German. No further restrictions apply to the inclusion based on the characteristics of the CGs. This approach ensures transparency in our adherence to established standards while allowing critical evaluation of the data handling and guideline application within these interventions.
- Comparison—studies must include a control group. The type of control treatment is not further specified, allowing for standard care or control interventions, providing flexibility for a wide range of comparative analyses. The studies must use a randomized design to ensure robust and unbiased results.
- Outcome—the primary outcomes are the effectiveness of CDSS-based interventions on different types of direct parameters of health and well-being. All other observed entities will be analyzed descriptively or through scoping methods. Indirect outcomes, such as compliance with guidelines or treatment acceptance, are not considered.
- Study design—studies must be randomized controlled trials.

The following language restrictions apply: non-English or non-German studies will be excluded.

### Databases and Literature Search

The literature search will be conducted using 2 primary databases: Web of Science (WoS; Clarivate), with the selection “all databases,” and Scopus (Elsevier). These databases collectively encompass approximately 160 million scientific references, including the MEDLINE database, which is particularly relevant for this review. On the basis of pilot search retrieval results, it was not considered necessary to expand the search to other databases (eg, CENTRAL) as the records would be duplicated with those found in WoS and Scopus.

### Search Expressions, Categorization, Relation to PICOS, and Search Terms

The search strategy is designed to capture relevant literature by applying specific search terms in alignment with the eligibility criteria. For the highest recall, only the necessary informative aspects of the eligibility criteria (Table 1) were implemented in the search expression (Textbox 1). The full search expressions are provided in Multimedia Appendix 1.

**Table 1.** Overview of search items.

Topic	Text word search	Medical Subject Headings search
Decision support systems	Decision support^a^ system^a^ Reminder system^a^ Recommender system^a^ Clinical decision rule^a^ Clinical decision algorithm^a^ Rule-based engine	Decision Support Systems, Clinical Reminder Systems
Guideline	Guideline^a^	Guidelines as Topic Treatment Guidelines as Topic
Clinical domain	Medic^a^ Clinic^a^	—^b^
Study type	Terms from the Haynes filter for therapeutic studies Combined with other terms for clinical studies	—

a Indicates truncation.

b Not applicable.

Textbox 1. **Search logic implemented in the search expression.**

The search expression implements the following search logic:

Clinical Decision Support System

AND

Guideline Intervention

AND

Randomized Controlled Trial Study Type

AND

(Medical OR Clinical) Domain

### Selection Process of Sources of Evidence

The inclusion criteria for this systematic review are derived from the eligibility criteria, focusing on studies involving human patients and the use of CDSS that are based on guideline knowledge, technically implemented, and aim to improve direct health outcomes (Textbox 2).

Textbox 2. **Inclusion and exclusion criteria.**

**Inclusion criteria**

- Population: human patients
- Intervention: clinical decision support systems (CDSS) with the following specific features:
  - Based on guideline knowledge
  - Including formalization of guidelines or technical implementation of the CDSS
  - Addressing health outcomes
- Outcome: direct health outcome
- Study type: randomized controlled studies
- Language: English or German

**Exclusion criteria**

- Population: no human patients
- Intervention: not a CDSS or not a guideline-based CDSS (ie, no technical information regarding formalization, implementation, or data processing)
- Study type: reviews, meta-analyses, guidelines, collections, or protocols
- Language: not English or German
- Missing information: no title or abstract, no data processing details, no technical implementation details, or no guideline formalization details in the CDSS
- Publication date: before 2010

### Searches and Integration

All papers are managed using Citavi (version 6.14; Lumivero) reference management software and categorized based on inclusion and exclusion criteria to facilitate the screening process after duplicates have been removed. A 3-round screening process, based on information including the abstract, and a final round based on the full text will be used by 3 authors (BNA, BG, and FS) to ensure a comprehensive and rigorous selection of studies for this systematic review. A fourth reviewer (MB) will review all included and undecided papers independently at every round. Discrepancies will be resolved through group discussion until consensus is reached.

### Data Collection Process and Data Items

Our data collection process is a structured and collaborative effort involving 4 team members. Initially, we gather data by copying and pasting relevant information from primary sources. Standardized data extraction forms will be developed to collect relevant information from each included study. The tables will capture key information aligned with the PICOS framework. If a paper has multiple medically relevant outcome parameters, we will capture them separately. The extracted data will be managed and organized using Microsoft Excel.

### Data Items

Data items are categorized into study details, guideline details, clinical decision support details, and clinical decision support guideline implementation details (Textbox 3). To ensure accuracy, data extraction will be cross-verified to identify discrepancies and improve data quality. Regular meetings will be held to review and refine the data collaboratively. Finally, we collectively edited and fine-tuned the data items for coherence and accuracy. This stepwise, collaborative approach ensured comprehensive and accurate data collection for our review.

Textbox 3. **Categories of data items extracted.**

**Study details**

- Setting—the context or environment where the study was conducted
- Period—the duration over which the study was carried out
- Target population—description of the target population
- Participants (sample)—number of individuals involved in the study and their demographics (age and gender)
- Intervention description—detailed description of the intervention being studied
- Control description—detailed description of the control or comparison group
- Outcome parameters
  - Main outcome parameter—the primary metric used to assess the effectiveness of the intervention
  - Secondary outcome parameter—additional metrics used to evaluate the intervention
- Results—a comprehensive description of the study findings, including numeric data

**Guideline details**

- Title—the title of the guideline used in the clinical decision support systems (CDSS)
- Source—the origin or organization that published the guideline
- Version—the specific version of the guideline
- Medical entities
  - Text—narrative description of medical terms
  - Coded (International Classification of Diseases–10 [ICD-10] and Systematized Nomenclature of Medicine–Clinical Terms [SNOMED CT])—coded representation of medical terms
- Authors—the individuals or group responsible for the guideline

**CDSS details**

- Name of the system—the name of the CDSS
- Developer, publisher, or company—the entity responsible for developing or publishing the system
- Source of information—the origin of the data or knowledge used in the system
- Description—a textual description of the system
- Classifications—the classifications used within the system, specifying which are applied
- Medical objectives
  - Text—narrative description of the system’s medical objectives
  - Coded (ICD-10 and SNOMED CT)—coded representation of the system’s medical objectives
- Implementation—how the system is technically implemented (eg, framework or databases) and integrated into clinical practice (hospital information system)
- Data processing (for analysis)—methods used for data processing and analysis
- Data
  - Source—the origin of the data
  - Type—the types of data used in the system, including data describing control groups and technical specifications of the CDSS. This includes patient data, clinical information, diagnostic data, and any other relevant data types that contribute to the functionality and effectiveness of the CDSS.
- Evaluation or validation—studies, methods, and results of the system’s evaluation and validation (if available, with references)

**Clinical decision support guideline implementation details**

- Description of integration—a detailed description of how the guideline is integrated into clinical practice
- Formalization
  - Text—narrative description of the formalization process
  - Classification—classification of the formalization, referring to relevant literature for specific categories

### Critical Appraisal of Individual Sources of Evidence

To allow readers to evaluate the risk of bias of the included studies, we use the revised “Risk of Bias” (version 2.0; Cochrane) tool for randomized trials [28]. For studies that were cluster randomized, we used the corresponding supplement [29]. Risk of Bias tool addresses five domains in individually randomized trials: (1) bias arising from the randomization process, (2) bias due to deviations from intended interventions, (3) bias due to missing outcome data, (4) bias in measurement of the outcome, and (5) bias in selection of the reported result. In cluster randomized trials, an additional domain is analyzed: bias arising from the identification or recruitment of individual participants within clusters [28]. Each study is assessed for risk of bias by 2 authors of the review; possible differences in assessment will be resolved by discussion. If necessary, arbitration will be provided by a third author.

### Effect Measures

The primary studies included in this review report various effect measures to quantify the outcomes. The primary effect measures used are odds ratios (ORs), mean differences, hazard ratios, and differences in proportions. Main effect measures are reported along with CIs and *P* values, where provided in the primary studies.

In cases where studies provide sufficient raw data, ORs with CIs and *P* values are calculated to ensure consistency across the analysis. However, for mean differences or differences in proportions, direct calculation of ORs is not feasible because of the nature of these measures. Instead, the interpretation focuses on the reported values. The diversity of effect measures used across the included studies reflects the multifaceted nature of the outcomes being investigated.

### Synthesis of Results

For a qualitative synthesis of results, a set of categories will be systematically derived to standardize the measurement of outcomes across different studies and data sources. These categories will be designed to capture the key elements that influence outcome assessment, ensuring a consistent approach to data collection, processing, and analysis. By defining these categories, the study aims to facilitate the accurate evaluation of outcomes and comparability of results across different studies and settings. A brief description of the possible categories is as follows:

- Patient characteristics—for example, outpatient and inpatient characteristics
- Patient data source system—indicates the electronic systems, platforms, or methods used to retrieve patient data, such as the electronic health record system
- Patient data source format—indicates the format of patient data and specifies whether standards (eg, Health Level 7, version 2) are used
- Patient data types—specifies the various types of patient data included, such as demographics, clinical notes, diagnoses, and laboratory results
- Electronic data capture (yes or no)—specifies whether electronic data capture methods are used for data collection
- Patient-reported outcome measures (yes or no)—indicates whether patient-reported outcome measures are included in the study
- Knowledge base—refers to the source from which the CDSS derives its knowledge, such as a rules database, medication database, or other specialized repositories

A quantitative synthesis (meta-analysis) of efficacy is not planned because of the significant heterogeneity of studies, investigated health conditions, interventions, and outcome measures.

## Results

On August 11, 2022, the initial search was conducted on WoS and Scopus. From a total of 6203 records, 1347 were removed prior to screening as duplicates, 2506 records were excluded during the first screening step, and 2291 were excluded during the second step. After that, 41 papers were excluded based on full-text review, of which 5 provided insufficient information, 1 were not conducted on human patients, 18 were not CDSS interventions, 3 were not guideline-based CDSS, 1 did not report on a health outcome, 1 had no control group, and 2 were protocols. In the fourth and final exclusion step, 5 papers were excluded for providing insufficient information on at least 2 of the following 3 criteria: implementation, data processing, or formalization. Furthermore, 3 papers were excluded because they had only borderline relevance or insufficient data, 1 was excluded for not being empirical, and 1 paper was excluded for not reporting a health outcome.

The 18 included studies were published in journals categorized as shown in Table 2.

In the clinical and specialty medicine category, 4 (22.2%) studies were included from pediatric journals, and 1 (5.6%) study each was included from journals in cardiology, critical care, infectiology, neurology, pain management, pharmacology, rheumatology, and obstetrics. As mentioned, the initial search was conducted on August 11, 2022; an update has been planned for April 2025.

**Table 2.** Number and proportion of studies included in the review, by journal category (N=18).

Journal category	Studies, n (%)
Clinical and specialty medicine	12 (66.7)
General medicine and public health	4 (22.2)
Medical informatics and digital health	2 (11.1)

## Discussion

### Anticipated Findings

This systematic review examines the effectiveness of guideline-based decision support systems on direct medical outcomes, including only studies in which the technical integration and algorithmic embedding of CGs have been described or can be inferred from secondary literature.

We hypothesize that formally grounded, technically sound CDSS connected to CGs have the potential to improve clinical outcomes. Nevertheless, their effectiveness may appear highly context dependent, varying across different settings and patient populations. We anticipate that evidence gaps will remain, particularly regarding long-term outcomes. Therefore, the review is likely to highlight the need for further research to strengthen the evidence base and validate the use of CDSS.

Due to the wide range of reported outcomes and the lack of comparability between the included studies, a meta-analysis is not planned. Taken together, the findings are expected to inform best practices and shape future research priorities, especially in standardizing the reporting on the formal representation of CGs and their technical integration in CDSS, as well as in comparing interventions across heterogeneous studies.

### Limitations

Potential limitations of this systematic review have been recognized. The primary challenge is the heterogeneity and quality of the existing literature. The included studies will vary widely in terms of design, population, CDSS, and outcome measures. This *heterogeneity* makes it challenging to draw definitive conclusions about the overall effectiveness of certain interventions and limits the generalizability of the findings. The review may be subject to *publication bias*, where studies with significant or positive results are more likely to be published and included in the review. This could skew the results toward more favorable outcomes, while the effectiveness of interventions with nonsignificant or negative findings might be underrepresented. We will address both issues by not including a quantitative analysis in the systematic review; instead, we will systematically derive a set of categories to standardize the measurement of outcomes across different studies and data sources.

The decision to include WoS, including MEDLINE, and Scopus as primary literature databases was driven by practical considerations and available resources. These databases cover most of the relevant literature in the medical domain. To ensure reproducible results and facilitate easy updates to this review, we refrained from including handsearching techniques (eg, forward and backward reference tracking) and gray literature. We address the potential bias introduced by this limitation with a very broad search strategy.

A significant bias may be introduced by the language restriction, which limits the review to studies published in English or German, potentially excluding important work not published in those languages. Further bias may be introduced by excluding studies of CDSS interventions implementing CGs that are not published by a discernible guideline-producing institution or are not published in English or German. This may affect studies from certain regions of the world; however, we consider the risk of significant bias to be small relative to the expected high heterogeneity and variability in reporting quality across studies. For full transparency, we will detail the criteria for study exclusion from the review in the final report, especially based solely on the language or quality of the implemented guideline.

### Conclusions

With this systematic review on the effectiveness of guideline-based decision support systems, we focus on studies in which the technical integration and algorithmic embedding of CGs have been described or can be inferred from secondary literature. Existing studies tend to focus on specific aspects or applications of CDSS, without providing a comprehensive analysis of how CGs are embedded within these tools. Consequently, there is a significant gap in understanding the methodologies used for guideline integration, the types of guidelines most commonly used, and the impact of these integrations on clinical outcomes.

This review aims to address this gap by providing a detailed analysis of existing research and identifying best practices, challenges, and areas for future investigation. It maps the current state of evidence on CDSS, with a particular focus on the integration of CGs.

## Supplementary material

10.2196/87006 Multimedia Appendix 1 Full search expressions for Web of Science and Scopus.

## Abbreviations

CDSS: clinical decision support systems
CG: clinical guideline
OR: odds ratio
PICOS: patient, intervention, comparison, outcome, and study design
PRISMA: Preferred Reporting Items for Systematic Reviews and Meta-Analyses
PRISMA-ScR: Preferred Reporting Items for Systematic Reviews and Meta-Analysis extension for Scoping Reviews
WoS: Web of Science
